# Early, Subclinical Hematological Changes Associated with Occupational Exposure to High Levels of Nitrous Oxide

**DOI:** 10.3390/toxics6040070

**Published:** 2018-11-21

**Authors:** Fatemeh Amiri, Masoud Neghab, Fatemeh Kargar Shouroki, Saeed Yousefinejad, Jafar Hassanzadeh

**Affiliations:** 1 Student Research Committee, Shiraz University of Medical Sciences, Shiraz 71645-111, Iran; amirif8484@gmail.com (F.A.); kargar_st@yahoo.com (F.K.S.); 2 Department of Occupational Health Engineering, Research Center for Health Sciences, Institute of Health, School of Health, Shiraz University of Medical Sciences, Shiraz 71645-111, Iran; yousefisa@sums.ac.ir; 3 Department of Clinical Epidemiology, School of Health, Shiraz University of Medical Sciences, Shiraz 71645-111, Iran; jafarabolhasan@yahoo.com

**Keywords:** inhalation anesthetic, hematological changes, occupational exposure

## Abstract

This study was undertaken to determine whether exposure of operating room personnel to inhalation anesthetics, nitrous oxide, isoflurane, and sevoflurane was associated with any hematological changes. This historical cohort study was performed in 2018 at a large public hospital in Shiraz, where 52 operating room personnel and 52 administrative staff were investigated. The blood sample was taken from all individuals for Complete Blood Count. Furthermore, demographic information was collected through questionnaires. Mean atmospheric concentrations of nitrous oxide, isoflurane, and sevoflurane, to which subjects were exposed, were 850.92, 2.40, and 0.18 ppm, respectively. The hematological parameters were within the normal range in both groups. However, the mean values of hemoglobin, hematocrit, mean corpuscular hemoglobin, mean corpuscular hemoglobin concentration, and red blood cell count in the exposed group were significantly lower than the control group. No significant differences were noted between the two groups as far as other hematological factors were concerned. These findings provide circumstantial evidence to further substantiate the notion that occupational exposure to inhalation anesthetics, under the exposure scenario explained in this study, is associated with subtle, subclinical, prepathologic hematological changes. Long-term consequence and ramifications of these effects require further investigation. The range of exposure levels to anesthetic gases in operating rooms.

## 1. Introduction

Healthcare centers are among the big and growing industries. Healthcare workers (HCWs) are exposed to different occupational hazards including waste anesthetic gases (WAGs) [1]. WAGs are trace amounts of inhalational anesthetic gases (nitrous oxide and halogenated anesthetics) that leak into the operating room air at the time of anesthetizing the patients [2]. According to estimates by OSHA, more than 200,000 HCWs in different environments including operating rooms, recovery rooms, dental clinics, and veterinary clinics are potentially exposed to WAGs [3].

Among inhalation anesthetics, nitrous oxide, isoflurane, sevoflurane, and desflurane are commonly used [4]. In Iran, nitrous oxide and isoflurane, extensively, and sevoflurane, to a lesser extent, are used in general anesthesia.

Nitrous oxide or laughing gas is the first modern anesthetic drug. It is a colorless gas and nonirritating with a sweetish smell [5,6]. The American Conference of Governmental Industrial Hygienists (ACGIH) and the National Institute for Occupational Safety and Health (NIOSH) recommended 50 and 25 ppm as the exposure limits for this gas, respectively [7,8].

Isoflurane and sevoflurane are halogenated ethers that are used as inhalation anesthetic agents. Isoflurane has a mildly pungent odor and irritates airways. Conversely, sevoflurane has a pleasant odor [9,10]. NIOSH has recommended an exposure limit (REL) of 2 ppm for sevoflurane and isoflurane [11].

HCWs are at the risk of acute or chronic toxicity during the use of anesthetic gases in the operating or recovery rooms [2,12,13,14,15,16].

Possible hematological effects of long term occupational exposure to isoflurane and sevoflurane have not been previously evaluated. However, this issue has been investigated, to a limited extent, in patients undergoing surgery and in experimental animals. For instance, Bozdogan et al. studied the hematological effects of isoflurane, sevoflurane, and desflurane in patients before and after undergoing minor surgery. No significant differences were noted between hematological factors prior to and after exposure to these anesthetic gases [17]. Additionally, the effects of ether, methoxyflurane, isoflurane, and carbon dioxide were studied in laboratory rats by Deckardt et al. The mean of red blood cell count, hemoglobin concentration, and hematocrit, only in the female rats exposed to isoflurane, were significantly lower than those of the control group [18].

Hematotoxicity has been reported following occupational exposure to nitrous oxide [19,20] as well as its application for anesthesia in patients [21,22] and in experimental animals [23,24].

For instance, Sweeney et al. studied the effects of nitrous oxide in 21 dentists and reported that occupational exposure to nitrous oxide concentrations ranging from 159 to 4600 ppm may cause noticeable bone marrow changes because of depression of vitamin B_12_ activity [19]. Similarly, blood dyscrasias have been reported following chronic occupational exposure to trace amounts of the nitrous oxide [20].

Amess et al. demonstrated that continuous exposure to 50% nitrous oxide and 50% oxygen for 24 h may cause megaloblastic hemopoiesis in patients undergoing surgery [21]. Likewise, Nunn et al. have reported megaloblastic hemopoiesis after intermittent short-term exposure to a mixture of nitrous oxide/oxygen (50/50) in a 33-year-old male patient [22].

In contrast, some studies have failed to demonstrate any blood dyscrasias following occupational exposure to nitrous oxide. For instance, De zotti et al. evaluated hematological factors of 61 operating room personnel exposed to anesthetic gases and 156 unexposed referent subjects. No significant differences were noted in the results of hemopoietic functions between both groups. Level of occupational exposure to nitrous oxide gas varied from 500 to 1275 ppm in theatres without scavenging [25].

Although the hematotoxic potentials of anesthetic gases have, to some degree, been studied in experimental animals and patients, this issue has not been extensively studied among subjects occupationally exposed to this compound.

This study was, therefore, undertaken to investigate, more thoroughly, whether exposure of operating room personnel to inhalation anesthetics—nitrous oxide, isoflurane, and sevoflurane—was associated with any significant hematological changes, as reflected in complete blood count (CBC) parameters.

## 2. Materials and Methods

### 2.1. Study Population and Design

This was a historical cohort study which was conducted at a large public hospital in Shiraz in 2018.

A total number of 52 operating room personnel (anesthesiologists, surgeons, surgical technicians, and anesthetic nurses) with current occupational exposure to inhalation anesthetics, nitrous oxide, isoflurane, and sevoflurane, as the exposed group and 52 unexposed referent subjects were investigated.

The criteria for selection of exposed subjects were: a history of at least one year of occupational exposure to anesthetic gases, lack of history of previous or present exposure (occupational or non-occupational) to other known hematotoxic agents, absence of self or family history of blood diseases (thalassemia, hemophilia, iron deficiency anemia, Fauvism, sickle cell anemia, thrombocytopenia, etc.), lack of history of taking hematotoxic drugs and lack of history of receiving blood for at least three months prior to the study.

The individuals in the control group were healthy sex and age matched employees, selected from nurses of inpatient wards and administrative departments of the hospitals. They satisfied the same inclusion criteria as for the exposed group except history of exposure to anesthetic gases.

All subjects completed and signed an informed consent form before entering the study. This study was approved by the ethics committee of Shiraz University of Medical Sciences (IR.SUMS.REC.1396.S388) and it was carried out according to the Helsinki Declaration of 1964 as revised in 2013 [26].

Demographic and occupational history of participants including age, sex, level of education, smoking habits (cigarette and nargile), intake of alcohol, work experience, mental and physical diseases, history of exposure to chemicals, and previous and current history of blood diseases was collected through questionnaires.

### 2.2. Sample Collection and Hematological Assays

Blood samples (2 cc) were taken from the antecubital vein of the subjects by a laboratory expert at their working place. The blood samples were transferred to CBC tubes containing EDTA as the anticoagulant, kept on ice packs in a cool box and immediately transported to the laboratory, where they were analyzed.

The blood parameters measured in this study were white blood cell (WBC) count, red blood cell (RBC) count, hemoglobin (Hb) level, hematocrit (HCT), mean corpuscular volume (MCV), mean corpuscular hemoglobin (MCH), mean corpuscular hemoglobin concentration (MCHC), red blood cell distribution width (RDW), platelet count (PLT), and mean platelet volume (MPV).

The tests were conducted by an automated Nihon Kohden hematology cell counter (serial number 11649, model Celltac-α, MEK-6410K), produced by Nihon Kohden Corporation, Tokyo, Japan in 2007.

The internal assessment of analytical quality was accomplished by regular measurement of reference samples (CELLCO Blood Control) obtained from an internal supplier, Arian Danesh, Co. (Tehran, Iran). All collected samples were blindly analyzed by one of the authors.

### 2.3. Exposure Assessment

USP grade isoflurane (Nicholas Piramal India Limited from Mumbai, Maharashtra, India) and USP grade sevoflurane (Abbott laboratories from Chicago, IL, USA) were utilized for preparation of standard solutions and calibration curve. The participants’ exposure to isoflurane and sevoflurane was determined in accordance with the OSHA method 103 [27]. The Anasorb 747 sorbent tubes (Cat. No. 226-81A; SKC Inc., Eighty Four, PA, USA) were connected to a calibrated low-flow pump (model SKC 210-1002, Blandford Forum, Dorset, UK) to collect personal air samples from the breathing zones of the participants. The sampling was performed at a flow rate of 50 mL/min. After air sampling, the sorbents were sealed and shipped to our laboratory for analysis. The adsorbed isoflurane and sevoflurane were extracted by 1.0 mL of carbon disulfide (CS_2_) (Merck KGaA, Darmstadt, Germany) containing 2 ppm of trichloroethylene (Merck KGaA, Darmstadt, Germany) as internal standard (IS) and then 1.0 µL was injected into a Varian CP-3800 gas chromatograph equipped with a flame ionization detector (GC–FID) (Varian Inc., Palo Alto, CA, USA) and a capillary column (CP-Sil 5 CB 30 m × 0.25 mm × 0.25 µm # CP8741 from Varian Inc., Palo Alto, CA, USA). The injector and detector temperatures were 250 °C and 300 °C, respectively. The initial oven temperature was 60 °C for 10 min, followed by an increase of 10 °C/min up to 100 °C, maintained for 2 min. Nitrogen was used as the carrier gas at the flow rate of 1.2 mL/min. Limits of detection for isoflurane and sevoflurane were 0.002 ppm and 0.003 ppm (in air volume), respectively.

The concentrations of nitrous oxide were measured using a portable infrared spectrophotometer (Bacharach model 3010, New Kensington, PA, USA) according to the NIOSH method 6600 [28]. The infrared spectrophotometer was calibrated by a nitrous oxide capsule 99.999% (SIG CO. Shiraz, Iran) according to the manufacturer instructions.

### 2.4. Data Analysis

Data were analyzed using an SPSS software version 21. Independent student’s *t*-test was used for comparing the mean of quantitative variables and chi-square was used for comparing the mean of qualitative variables. Furthermore, multiple linear regression analysis was used to control the confounding variables such as age, sex, body mass index (BMI), etc. *p*-values of <0.05 were considered significant.

## 3. Results

Demographic characteristics, length of exposure or employment and smoking habits of the studied groups are presented in Table 1. According to the results, no statistically significant differences were noted between the two groups as far as these variables were concerned (*p*-value > 0.05). Length of exposure of the participants of this study to inhalation anesthetic was about 8 h per day.

The geometric mean concentration of nitrous oxide, isoflurane, and sevoflurane in the air of studied operating rooms are presented in Table 2.

Table 3 shows that the results of the complete blood count (CBC) in exposed and unexposed groups were within the normal range. However, statistical analysis revealed that the mean of hemoglobin (Hb), hematocrit (HCT), mean corpuscular hemoglobin (MCH), mean corpuscular hemoglobin concentration (MCHC), and red blood cell (RBC) count in the exposed group were significantly lower than the control group (*p*-value < 0.05).

No significant differences were observed between exposed and unexposed groups for white blood cell (WBC) count, platelet (PLT) count, red blood cell distribution width (RDW), mean corpuscular volume (MCV), and mean platelet volume (MPV) (*p*-value > 0.05).

Multiple linear regression analysis, including age, sex, and body mass index (BMI), in the model revealed that after adjusting for these major confounders, there were statistically significant associations between exposure to anesthetic gases and changes in most CBC parameters (Table 4).

In Table 4, Beta is the average unit of changes in each of CBC parameters of the exposed subjects compared to the unexposed group.

## 4. Discussion

The present study was undertaken to ascertain whether occupational exposure to inhalation anesthetics is associated with any significant hematological changes among operating room personnel of a large public hospital in Shiraz.

None of the subjects had any medical or family history of blood diseases such as fauvism, thalassemia, hemophilia, cycle cell anemia, thrombocytopenia, and anemia. Moreover, there were no significant differences between the two groups with respect to demographic variables, smoking habits, and length of employment.

The mean concentration of nitrous oxide was 851 ± 919.78 ppm, which was 17- and 34-fold higher than the existing exposure limits of 50 and 25 ppm recommended by ACGIH and NIOSH for this gas, respectively [7,8].

This concentration while, quantitatively, is similar to those reported approximately three decades ago [19,29], it is significantly higher than those reported in recent years. For instance, Baek et al. assessed occupational exposure to nitrous oxide before and during operation. They reported that nitrous oxide concentrations in the operating rooms varied from 14.39 to 282.86 ppm [30]. Similarly, Latiff et al. measured the concentration of nitrous oxide in operating rooms. They compared two modes of ventilation (laryngeal mask airway (LMA) and endotracheal tube (ETT)). The mean concentrations of nitrous oxide in operating rooms were 14.7 and 17.4 ppm for ETT and LMA modes, respectively. Concentration of nitrous oxide in the morning, before use of nitrous oxide, was less than 2 ppm [31].

The finding of an unusual, very high mean atmospheric concentration of nitrous oxide in our study is likely to be due to the absence of proper ventilation systems in the operating rooms, lack of proper scavenging systems, leaks from anesthesia face masks during the administration of the anesthetic gases to the patients, leaks from nitrous oxide cylinders, lack of regular check for detecting gas leakage from anesthetic machines, inappropriate work practices such as starting anesthetic gas flow before applying a mask on the patient’s face or closing the anesthetic gas flow after removing a face mask and poorly fitted face masks.

The mean concentration of isoflurane was 2.40 ± 0.86 ppm, which was slightly higher than the recommended TLV of 2 ppm for this compound [11]. Conversely, mean concentration of sevoflurane was 0.18 ± 0.14 ppm, which was below the recommended TLV of 2 ppm for this gas.

Given the above, it would reasonable to assume that nitrous oxide was the main source of pollution in the operating room and an important health threat for exposed personnel.

The findings of the current study showed that hemoglobin (Hb), hematocrit (HCT), mean corpuscular hemoglobin (MCH), mean corpuscular hemoglobin concentration (MCHC), and red blood cell (RBC) count were significantly lower in the exposed group than in the control group. (Table 3).

The results of multiple linear regression analysis showed that after adjusting for important confounders (age, sex, and BMI) statistically significant negative associations exist between exposure to anesthetic gases and Hb, HCT, MCH, MCHC values, and RBC count, in that, exposure to anesthetic gases resulted in 1.05, 2.17, 1.04, 0.89, and 0.21 units of decrease in these parameters, respectively.

Although these findings may be attributed to exposure to a mixture of nitrous oxide, isoflurane, and sevoflurane, the role of nitrous oxide in this scenario is thought to be more prominent. First, because the average concentrations of isoflurane and sevoflurane to which subjects were exposed were very low or below their corresponding TLVs. Second, nitrous oxide was the main source of pollution in the operating rooms and the average concentration of nitrous oxide was 17- and 34 fold higher than its recommended TLV by ACGIH and NIOSH, respectively [7,8].

Third, no conclusive evidence exists to support the notion that isoflurane and sevoflurane have the propensity to induce hematotoxicity in humans [16,32,33].

Fourth, exposure to nitrous oxide has been shown to be associated with hematotoxicity.

For instance, Ames et al. confirmed megaloblastic change in bone marrow of patients following prolonged ventilation with 50% nitrous oxide in oxygen for 24 h [34]. Similarly, Peric et al. studied the effects of chronic occupational exposure to high concentrations of nitrous oxide and halothane on CBC parameters in 21 anesthetic staff before the holiday and after three weeks of holiday. Hematological parameters included WBC differential, hemoglobin, hematocrit, and erythrocyte count. They showed that chronic exposure to very high concentrations of nitrous oxide and halothane was associated with significant reductions in CBC parameters of anesthetic staff [35].

It has been proposed that nitrous oxide oxides the Co^+^ in vitamin B_12_ to Co^3+^, resulting in_._ inhibition of methionine synthetase, vitamin B_12_ deficiency and megaloblastic anemia [33,36]. In this regard, Sweeney et al. studied the effects of nitrous oxide in 21 dentists and reported that occupational exposure to nitrous oxide concentrations ranging from 159 to 4600 ppm may cause noticeable bone marrow changes because of depression of vitamin B_12_ activity [19].

Despite the presence of an association between occupational exposure to nitrous oxide and hematological changes, some investigators have failed to demonstrate that occupational or non-occupational exposure to nitrous oxide was associated with changes in blood parameters. For instance, Krajewski et al. compared 95 female surgical nurses with 90 female nurses. The former had previous exposure to anesthetic gases (nitrous oxide, isoflurane, sevoflurane, or halothane). No significant differences were noted in the results of hematological parameters between both groups. Mean levels of nitrous oxide in the respiratory area of nurses ranged from 19.89 to 834.39 ppm depending on the ventilation system [37]. Similarly, Salo et al. compared healthy operating room personnel (*n* = 10) exposed to nitrous oxide with a control group (*n* = 12). They did not observe any significant differences between the hematological factors of both groups. The mean nitrous oxide concentrations in the operating rooms varied from 155 to 860 ppm [29]. In another study, authors reported that hematotoxicity of nitrous oxide occurs at anesthetic concentrations but not at lower concentrations to which operating room personnel are exposed [38]. Abascall et al. investigated the effects of occupational exposure to nitrous oxide on blood parameters in 30 healthy full-time midwives. Median time weighted average of nitrous oxide was 41 (10–547 ppm). No signs of megaloblastic changes were seen in exposed personnel before and after vacation [39].

Although the exact cause of these inconsistencies and discrepancies between the researchers’ observations are not clear, differences in cumulative exposure concentrations, type of exposure (occupational or non-occupational), duration of exposure, sample size, type of statistical analyses (univariate or bivariate), control or lack of control of confounders, and preexisting medical conditions and history of exposure or current co-exposure to other hematotoxic agents may explain, at least in part, these differences. In line with this proposition Smith has shown that the side effects of nitrous oxide are dependent on duration, intensity and pattern of exposure [40].

Exposure to nitrous oxide is expected to be associated with megaloblastic anemia as a result of vitamin B_12_ deficiency. In this condition, the red blood cells are larger than the normal RBCs with a MCV > 100 fL and anisocytosis [41], a phenomenon which was not observed in this study. This apparent inconsistency deserves comment. RDW and MCV are two useful indicators for evaluating megaloblastic anemia [42]. Despite the fact that the presence of high MCV and anisocytosis suggest megaloblastic anemia, these changes may not necessarily be seen in this abnormality [41], particularly at the early stages. Additionally, MCV and RDW are not specific and sensitive hematological biomarkers for diagnosing vitamin B_12_ deficiency-induced megaloblastic bone marrow change [43,44,45]. Similarly, in the megaloblastic anemia associated with vitamin B_12_ deficiency, the large oval red blood cells appear in the bone marrow, not in the systemic circulation [46].

Because of the inherent limitations of the historical cohort studies and the small sample size of the study, it is not possible to establish a cause and effect relationship. Therefore, one might argue that the significant hematological changes noted in the operating room personnel as compared to their unexposed counterparts, may not necessarily be attributed to the exposure to the anesthetic gases. While from an epidemiological point of view, this scientific skepticism is difficult to refute, the authors maintain that the following lines of circumstantial evidence indicate that the observed effects are very likely to be related to exposure to the anesthetic gases.

First, none of the exposed subjects had any personal or family history of hematologic diseases or preexisting medical conditions at the beginning of their employment.

Second, apart from nitrous oxide, the exposed group had no history of exposure to other chemicals known to cause disorders of the hematopoietic system before their employment or during the course of their employment in the operating rooms.

Third the hematological indices of exposed group were significantly different from their unexposed counterparts, lending some support in favor of a link between exposure and outcome

Fourth, after adjusting for important confounders, the associations between exposure to nitrous oxide and reduction in CBC parameters remained statistically significant, implying that the observed effects are work-related.

## 5. Conclusions

These findings provide circumstantial evidence to support the notion that occupational exposure to inhalation anesthetics (nitrous oxide), under the exposure scenario explained in this study, is associated with subtle, subclinical, prepathologic hematological changes. Additional prospective cohort studies with larger sample sizes and sufficient follow up among subjects occupationally exposed to inhalation anesthetics are clearly required to further substantiate these findings and to assess possible long-term consequence and ramifications of these effects.

The very high atmospheric concentration of nitrous oxide observed in this study highlights the need and calls for urgent engineering and administrative prevention measures. The use of appropriate scavenging equipment, improved artificial ventilation systems, monitoring of nitrous oxide level in operating rooms by nitrous oxide analyzer, administration of intravenous anesthetic agents instead of anesthetic gases whenever possible, in service educational programs for the operating room staff (hazards of anesthetic gases, procedures to minimize exposure, safe work practices, checking for leaks in gas lines of the anesthesia machines, recognition of damaged equipment, proper maintenance of anesthesia devices, starting gas flow after applying a mask on the patient’s face, etc.) and periodic medical examinations should be strictly enforced to eliminate or reduce exposure and to protect the health of employees against the toxic effects of anesthetic gases in general and nitrous oxide, in particular.

## Figures and Tables

**Table 1:** Demographic characteristics of the study participants (mean ± SD).

Demographics Data	Exposed Group (*n* = 52)	Unexposed Group (*n* = 52)	*p*-Value
Age (yr)	34.19 ± 5.82	33.73 ± 6.84	0.710 *
Weight (kg)	69.74 ± 12.12	70.67 ± 12.36	0.702 *
Height (cm)	168.04 ± 9.19	170.63 ± 8.56	0.139 *
BMI (kg/m^2^)	24.59 ± 3.15	24.18 ± 3.41	0.533 *
length of exposure/employement (yr)	10.79 ± 5.63	8.62 ± 6.53	0.073 *
Number of smokers (%)			0.55 ^†^
Yes	1(2)	2(4)	
No	51(98)	50(96)	
Marital status (%)			0.126 ^†^
Married	41(79)	34(65)	
Single	11(21)	18(35)	

* Independent sample t test. ^†^ Chi-square test.

**Table 2:** Mean air concentration of anesthetic gases (ppm) for exposed individuals.

Anesthetic Gas	Number of Samples	Mean	SD	Median	Minimum	Maximum
Nitrous oxide	620	850.92	919.78	471	10	2895
isoflurane	35	2.40	0.86	2.52	0.49	4.15
sevoflurane	35	0.18	0.14	0.13	0.01	0.59

SD: standard deviation.

**Table 3:** Comparison of complete blood count tests between exposed and unexposed groups (mean ± SD).

Indices (Units)	Exposed Group (*n* = 52)	Unexposed Group (*n* = 52)	*p*-Value
WBC (mm^3^ blood × 10^3^)	6.79 ± 2.58	6.11 ± 1.23	0.095
RBC (mm^3^ blood × 10^6^)	4.67 ± 0.46	4.88 ± 0.58	0.048 *
Hb (g/L)	13.17 ± 1.74	14.21 ± 1.27	0.001 *
Hct (%)	39.09 ± 4.14	41.21 ± 4.53	0.017 *
MCV (fL)	83.86 ± 5.69	84.87 ± 5.02	0.348
MCH (pg)	28.22 ± 2.66	29.21 ± 1.56	0.021 *
MCHC (g/L)	33.61 ± 1.47	34.49 ± 1.77	0.007 *
PLT (mm^3^ blood × 10^3^)	213.36 ± 40.05	219.71 ± 55.68	0.517
RDW (%)	12.67 ± 0.87	12.85 ± 1.19	0.409
MPV (fL)	5.82 ± 0.67	6.08 ± 0.98	0.166

* independent sample *t*-test, *p* < 0.05.

**Table 4:** Association between exposure to anesthetic gases and changes in the complete blood count (CBC) parameters using the linear regression analysis.

Indices *	Beta	SE	CI		*p*-Value
			Lower	Upper	
WBC (mm^3^ blood × 10^3^)	0.22	0.47	−0.72	1.16	0.64
RBC (mm^3^ blood × 10^6^)	−0.21	0.08	−0.38	−0.05	0.01 ^†^
Hb (g/L)	−1.06	0.19	−1.45	−0.67	0.0001 ^†^
Hct (%)	−2.18	0.64	−3.44	−0.91	0.001 ^†^
MCV (fL)	−1.01	1.08	−3.16	1.13	0.34
MCH (pg)	−1.03	0.44	−1.9	−0.16	0.02 ^†^
MCHC (g/L)	−0.89	0.3	−1.5	−0.29	0.004 ^†^
PLT (mm^3^ blood × 10^3^)	−1.1	11.2	−23.4	21.08	0.91
RDW (%)	−0.08	0.24	−0.55	0.38	0.71
MPV (fL)	−0.23	0.17	−0.56	0.09	0.16

* Data for reference group were used as baseline values. ^†^ Significantly different (linear regression analysis, *p* < 0.05). SE, standard error.
